# Experimental natural transmission (seeder pig) models for reproduction of swine dysentery

**DOI:** 10.1371/journal.pone.0275173

**Published:** 2022-09-27

**Authors:** Juan Parra-Aguirre, Roman Nosach, Champika Fernando, Janet E. Hill, Heather L. Wilson, John C. S. Harding

**Affiliations:** 1 Department of Large Animal Clinical Sciences, Western College of Veterinary Medicine, University of Saskatchewan, Saskatoon, SK, Canada; 2 Department of Microbiology, Western College of Veterinary Medicine, University of Saskatchewan, Saskatoon, SK, Canada; 3 VIDO/Intervac, University of Saskatchewan, Saskatoon, SK, Canada; Plum Island Animal Disease Center, UNITED STATES

## Abstract

Swine dysentery is causally associated with *Brachyspira hampsonii* and *B*. *hyodysenteriae* infection. Given the importance of transmission models in understanding re-emergent diseases and developing control strategies such as vaccines, the objective of this experiment was to evaluate two experimental natural transmission (seeder pig) models in grower pigs, each with 24 animals. Seeder pigs were intragastrically inoculated using broth cultures of either *B*. *hampsonii* strain 30446 (genomovar II) or *B*. *hyodysenteriae* strain G44. In trial 1, three seeder pigs were placed into two pens containing nine susceptible contact pigs creating a 1:3 seeder:contact ratio. This was sufficient to achieve natural *B*. *hampsonii* infection of 13/18 (72%) contact pigs, however, the incidence of mucoid or mucohemorrhagic diarrhea (MMHD) in contact pigs differed significantly between pens (4/9 versus 9/9; *P* = 0.03). In trial 2, eight seeder pigs inoculated intragastrically with *B*. *hampsonii* did not develop MMHD but when re-inoculated with *B*. *hyodysenteriae* 14 days later, all developed mucohemorrhagic diarrhea within 13 days of re-inoculation. Two seeder pigs were placed into each of 4 contact pens each containing 4 pigs. This 1:2 seeder:contact ratio resulted in natural infection of 14/16 (87%) contact pigs with incubation period ranging from 9–15 days. There were no significant differences among pens in incubation period, duration, clinical period or severity of diarrhea. These trials demonstrated that a 1:2 seeder:contact ratio with groups of six grower pigs per pen sustained natural transmission of *B*. *hyodysenteriae* G44 with greater consistency in the incidence of MMHD among pens compared to a *B*. *hampsonii* 30446 transmission model using 1:3 seeder:contact ratio in pens of 12. Understanding why *B*. *hampsonii* intragastric inoculation failed in one experiment warrants additional research.

## Introduction

Swine dysentery (SD) is a bacterial disease caused by anaerobic, Gram-negative spirochetes of the *Brachyspira* genus. Historically, SD has been causally associated with *B*. *hyodysenteriae*, but newly identified strongly beta-hemolytic species, *B*. *hampsonii* and *B*. *suanatina*, also share the same fecal-oral transmission route with indistinguishable signs of mucoid or mucohemorrhagic diarrhea (MMHD) and colitis in grower and finishing pigs [1]. SD results in economic losses to the swine industry due to increased feed conversion rate, medication costs, growth reduction, and elevated mortality rates [2]. Since the early 1990s, improved management systems led to reductions in SD in North America [3], however, outbreaks of dysentery-like disease in late 2000’s signaled its re-emergence [4].

Non-pathogenic *Brachyspira* are normal inhabitants of the large intestine of healthy animals [5], but the colonization of pathogenic *Brachyspira* species resulting in clinical and pathological manifestations seems to depend on the number of bacteria, susceptibility of the host [6], and the interaction with other specific anaerobes [7]. Characteristic clinical signs of SD include chronic intermittent diarrhea ranging from watery to muco-hemorrhagic, and marked inflammation restricted to the large intestine of pigs, characterized by a fibrinonecrotic typhlocolitis with diffuse mucosal hyperemia and crypt elongation [8]. Pigs of all ages are susceptible to SD however, the infection occurs more frequently during the grower-finisher phase.

Experimental models of swine dysentery typically rely on direct inoculation of the bacteria to induce the disease in pigs with variable success due to variation in *Brachyspira* challenge dose, volume, inoculation method, diet, host genetics and administration route (sustained oral exposure versus a bolus or intragastric dose). Natural transmission or seeder pig models for SD have been used to evaluate strain pathogenicity and vaccine efficacy and are advantageous in terms of attempting to mimic SD transmission on commercial farms. Past attempts have used either inoculated animals or diarrheic animals from infected farms as the source of seeder pigs [9,10]. However, protocols are sparse and results can be variable due to factors including *Brachyspira* strain, inoculation dose, housing details (animal density, flooring type), and seeder to contact ratio. Thus, the purpose of this study was to develop a reproducible natural transmission model that could be used for the evaluation of a vaccine against strongly hemolytic *Brachyspira* spp.. The results of two infection and natural transmission models in grower pigs, using either *B*. *hampsonii* (trial 1) or *B*. *hyodysenteriae* (trial 2) as inoculum, are presented.

## Material and methods

### Ethics statement

These trials were designed and conducted in accordance with the Canadian Council for Animal Care and approved by the University of Saskatchewan Animal Research Ethics Board (Protocol #20180046) and ARRIVE guidelines (Animal Research: Reporting In Vivo Experiments). Both trials were conducted in biocontainment level 2 facilities at the University of Saskatchewan by experienced research staff who had successfully completed a mandatory UACC Ethics Course in Farm Animals. The clinical state of the animals was evaluated twice daily, and monitored according to Humane Intervention Points (HIP) established prior to ethics approval (S4 Table). To enable assessment of measurement of disease duration, endpoints were at a fixed time after all or a majority of pigs had recovered, except when wellbeing was unacceptably compromised. In such cases when welfare is compromised, euthanasia is performed within 6–12 hours, as soon as the necropsy team can be assembled. The latter included lack of responsiveness, extreme hyperemia or cyanosis of skin, increased respiratory rate and marked abdominal effort (dyspnea), persistent MMHD with moderate loss of body condition, rectal temperature above or below 41.5 ⁰C and 35 ⁰C. Euthanasia was conducted by cranial captive bolt followed by exsanguination.

### Experimental design and procedures

#### Trial 1. B. hampsonii strain 30446 (genomovar II) natural transmission model using a 1:3 seeder:contact ratio

Twenty-four healthy, seven-week-old, crossbred pigs (22 males, 2 inadvertent females) were obtained from the Prairie Swine Centre Inc. (Saskatoon, Canada) confirmed free of SD based on clinical signs and history, absence of antibiotics in feed and water, and fecal cultures performed prior to the trial initiation. Pigs were individually identified at arrival, weighed, and blocked by weight into two 8 × 10 ft (2.44 × 3.05 m) open-sided pens each containing 12 pigs. Pens had solid floor with rubber mats covering 25% of the pens without additional bedding. *Ad libitum* water and a non-medicated custom swine dysentery diet (#19277) (S1 Table) were provided for the duration of the trial (acclimation period plus 34 days post-inoculation). Pigs acclimated to their new diet and room environment for 8 days prior to inoculation. Pigs with episodes of mild diarrhea during the acclimation period were tested by submission of feces for *Brachyspira* culture (described below) and by submission of feces to the Prairie Diagnostic Services Inc. for routine diagnostic testing (*Escherichia coli* and *Salmonella* by culture, porcine enteric coronaviruses (deltacoronavirus, porcine epidemic diarrhea virus, transmissible gastroenteritis virus) by PCR). The day prior to inoculation, three animals from each pen (seeder pigs) were selected using a random number generator and transferred to two 4 × 6 ft (1.21 × 1.82 m) open-sided inoculation pens within the same room.

On 0, 1 and 2 days post-inoculation (DPI) seeder pigs (n = 6) were sedated intramuscularly (IM) with ketamine (8 mg/kg, Ketalean, Vetoquinol), acepromazine (0.1 mg/kg, Acevet 25, Vetoquinol), and xylazine (2 mg/kg, Rompun, Bayer) and inoculated intragastrically using an 18 French foal feeding tube while lying in lateral recumbency. Fifty mL of a 24-hour broth culture of *B*. *hampsonii* strain 30466 P13 was administered at doses 8.85 × 10^8^, 3.03 × 10^8^, and 1.38 × 10^9^ genome equivalents (GE)/mL (D0-2, respectively), followed by 30 mL of sterile PBS (0.1M, pH 7.0). Before each inoculation, seeder pigs were fasted for 15 hours to decrease gastric transit time. Water was withheld about 2 hours before seeder pig inoculation to empty the stomach of fluid. Water was made available immediately after inoculation and pigs re-fed when they could stand unassisted. Seeder pigs were reintroduced with their previous pen mates at 5 DPI establishing a 1:3 seeder:contact ratio. Contact pigs that remained in their original pens were not fasted, sedated nor inoculated. To avoid cross contamination between seeder and contact pigs prior to re-introduction, boot baths were installed at the front of each pen and human traffic in and out of the pens were restricted from 0 to 5 DPI. Rectal swabs were collected from seeder pigs on -6, -2, 0, DPI and daily from 5 to 12 DPI. Rectal swabs from contact pigs were collected on -6, 0, 7, 14, 21 DPI.

#### Trial 2. B. hampsonii strain 30446 followed by B. hyodysenteriae strain G44 natural transmission model using a 1:2 seeder:contact ratio

Trial 2 followed similar procedures as trial 1, with several major differences noted below. Pigs were purchased from the same farm following pre-screening of five of the oldest nursery pigs for strongly or weakly hemolytic *Brachyspira* strains using rectal swabs cultured on BJ selective medium. Twenty-four healthy, six-week-old, crossbred barrow pigs, originating from five different litters (4 pigs per litter) were assigned to four 6’ × 6’ (1.82 × 1.82 m) open-sided pens blocked by litter of origin. One day prior to inoculation (-1 DPI), eight seeder pigs (all from the same litter except one) were moved into four inoculation pens (2 pigs per pen) and intragastrically inoculated on three consecutive days (D0 to 2) with 50 mL of a 24-hour broth culture of *B*. *hampsonii* strain 30446 at 8.77 × 10^8^, 5.25 × 10^8^, and 6.88 × 10^8^ GE/mL per day. On 5 DPI, the seeder pigs were reintroduced into their original pens in a 1:2 seeder:contact transmission ratio for each pen. Unexpectedly, none of the seeder pigs developed clinical signs consistent with SD. The previously assigned seeder pigs were transferred again into inoculation pens and intragastrically re-inoculated on 14 DPI (hereafter termed as 0 days post reinoculation (DPRI)) for three consecutive days with 50 mL of a 24-hour broth culture of *B*. *hyodysenteriae* strain G44 at 8.94 × 10^8^, 8.04 × 10^8^, and 3.38 × 10^9^ GE/mL per day. On 5 DPRI, the re-inoculated seeder pigs were reintroduced with their previous pen mates in a 1:2 seeder:contact transmission ratio until termination of the study (28 DPRI). The contact group was not inoculated. Fecal samples of seeder pigs were collected free fall or digitally on -6, -2, 0, 5, 8, 13 DPI. Fecal samples from contact pigs were collected on -6, -2, 0, 5, DPI. Additionally, fecal samples of all pigs were collected on 5 and 15 DPRI. The trial duration was 49 days total (7 days acclimation, 14 days post-B. hampsonii inoculation, 28 days post-B. hyodysenteriae inoculation).

### General and laboratory procedures

#### Source of B. hampsonii 30446 and B. hyodysenteriae G44

The source of *B*. *hampsonii* strain 30446 was described by Rubin [11]. Briefly, *B*. *hampsonii* strain 30446 originated from a frozen stock (-80°C) of colonic and cecal mucosa samples of clinically affected 13-week-old pigs from a porcine reproductive and respiratory syndrome virus (PRRSV) negative farm. The isolate was subject to 13 passages on JBS broth culture for purification purposes, aliquoted and frozen for future use. *B*. *hyodysenteriae* strain G44 was generously donated by Boehringer Ingelheim Vetmedica (St. Joseph, MO) and was propagated in JBS broth and aliquoted. An aliquot of each inocula was assessed for purity using *nox* PCR and sequencing. The concentrations of *B*. *hampsonii* strain 30446 and *B*. *hyodysenteriae* G44 in the inoculum were determined by quantitative PCR [11,12].

#### Preparation of inocula

Frozen, purified isolates of either *B*. *hampsonii* strain 30446 or *B*. *hyodysenteriae* strain G44 were cultured in JBS broth [11] and anaerobically incubated in sterile jars using gas-packs (Anaerogen TM 2.5 L, Thermo Scientific® Oxoide Sachet) at 37°C for 24 hours on a magnetic stirrer. After each 24-hours of incubation, a 1:9 broth culture dilution (v/v) in JBS broth was prepared scaling up from 10 mL to 500 mL with a concentration target of 1× 10^8^ to 10^9^ GE/mL.

#### Brachyspira culture

To monitor fecal shedding of *Brachyspira*, feces, fecal swabs and intestinal tissues were cultured on BJ media [13] in an anaerobic chamber (Whitley DG250®; 5% carbon dioxide, 10% hydrogen, balance nitrogen) at 41 ± 1°C and semi-quantitatively evaluated using the quadrant method. Any strong hemolysis in any quadrant was considered a positive result. Plates were evaluated at 48 and 96 hours of incubation.

#### DNA extraction and PCR

DNA from fecal samples (200 mg) and colonic tissue (20 mg) collected at termination was extracted using the QIAmp DNA stool mini kit (Qiagen Inc., Toronto, ON) or DNEasy blood and tissue kit (Qiagen Inc.), respectively. Fecal swabs were immersed in 1 ml InhibiTEX buffer for 1 minute, then vortexed and 200 μl used for extraction using a commercial kit (QIAmp DNA Stool Mini kit) as per the manufacturer’s instructions. Detection and identification of *Brachyspira* spp. from swabs, feces and colonic tissue were accomplished by amplifying the NADH-oxidase (*nox*) gene followed by sequencing amplicons [11,14]. *B*. *hampsonii* strain 30446 and *B*. *hyodysenteriae* strain G44 were quantified using SYBR green real-time qPCR on a Bio-Rad MyiQ thermocycler. The primers used for *B*. *hampsonii* 30446 were JH0224 (5’-TCG CTA AAT TAT TCC AAC AAG GA-3′) and JH0225 (5′-AAA CGC ATT TCT ATT CCA GCA-3′). The primers used for *B*. *hyodysenteriae* were JH0073 (5’-AGT GAA ATA GTT GCT CAT ATC AAA T-3′) and JH0074 (5′-GCA TCA CTG ATT AAA GAA CCA AT-3′)). Primers (400 nM) were mixed with 2 μL of template DNA and 1× SYBR Green Supermix (Bio-Rad Laboratories (Canada) Ltd., Mississauga, ON) in a final volume of 25 μL with each assay performed in duplicate. Every plate for qPCR analysis included a no-template control and plasmid standard curve ranging in concentration from 10^0^ to 10^7^ copies/reaction. Samples producing Cq values greater than the Cq of the lowest concentration standard were reported as detectable but non-quantifiable (DNQ).

Results were reported as logarithm base 10 per unit (mL for broth culture or gram for feces).

#### Clinical assessments and necropsy

All pigs were clinically assessed twice daily pre- and post-inoculation for general illness and diarrhea following the Human Intervention Point (HIP) checklist. Fecal consistency (FCS) was scored as: 0 = normal/formed, 1 = wet cement/loose cow pie, 2 = runny/ watery, 3m = mild mucoid, 3s = severe mucoid, 4m = mild bloody, 4s = severe bloody diarrhea. All pigs were humanely euthanized by cranial captive bolt and exsanguination at the scheduled termination day, unless euthanized earlier for humane reasons.

The gastrointestinal tract was examined post-mortem and a qualitative assessment of gross pathology was made. A 3 × 0.5 cm piece of colonic tissue from the apex spiral colon was collected from each animal. Tissues collected from each pig were cultured and subjected to *nox* PCR to detect the presence of either *B*. *hampsonii* strain 30446 or *B*. *hyodysenteriae* G44.

### Statistical analyses

The incidence of mucoid and/or hemorrhagic diarrhea (FCS ≥3), incubation period (sum of days from challenge to FCS ≥3), severity of diarrhea (sum of all FCS post-inoculation), clinical period (sum of days from onset of FCS ≥2 to recovery with FCS ≤2) and duration of MMHD (sum of days with FCS ≥3) were assessed for each trial. FCS ranked mild and severe were converted to numeric values (3m = 3.0, 3s = 3.5, 4m = 4.0, and 4s = 4.5). Non-parametric tests were used for statistical analysis due to the small sample size of the treatment groups and non-normal data distribution. To analyze potential differences in the incidence of MMHD between pens, a Fisher’s exact test was used. To analyze differences of the incubation period between pens, a Kaplan Meier Survival analysis was used. To assess pen differences in the duration of MMHD, clinical period and severity, Mann Whitney test or Kruskal Wallis followed by Dunn’s test, if significant, were used for trials 1 and 2, respectively. For trial 2, the effect of litter on the incidence of MMHD in contact pigs was analyzed using a Kaplan Meier Survival analysis. Potential litter effects in the duration of MMHD, clinical period and severity were assessed using Kruskal Wallis test. Spearman’s rank-order correlation was used to evaluate the strength of the relationship between fecal and culture scores, according to the following criteria: 0.9 to 1.00 very high; 0.70 to 0.90 high; 0.5 to 0.70 moderate positive; 0.30 to 0.50 low; 0.00 to 0.30 negligible correlation [15]. Incidence, duration of MMHD, clinical period and severity analyses were performed using Stata ® (version 15.1). Incubation period analyses were performed using the R environment for statistical computing (R version 3.5.3). Significant differences were established *a priori* at alpha ≤ .05.

## Results

### Trial 1. B. hampsonii strain 30446 natural transmission model using a 1:3 seeder:contact ratio

During the acclimation period, an acute, wet cement-type diarrhea (FCS = 1) was observed in 22/24 (91.6%) pigs lasting for 1 to 3 consecutive days before resolving without antibiotic treatment by -2 DPI. Adjunct diagnostic testing confirmed the presence of *Escherichia coli* (STb: AIDA-1 positive) and *Salmonella enterica* serovar Ohio, and ruled out the presence of *Brachyspira* spp. and porcine enteric coronaviruses.

**Fig 1:**
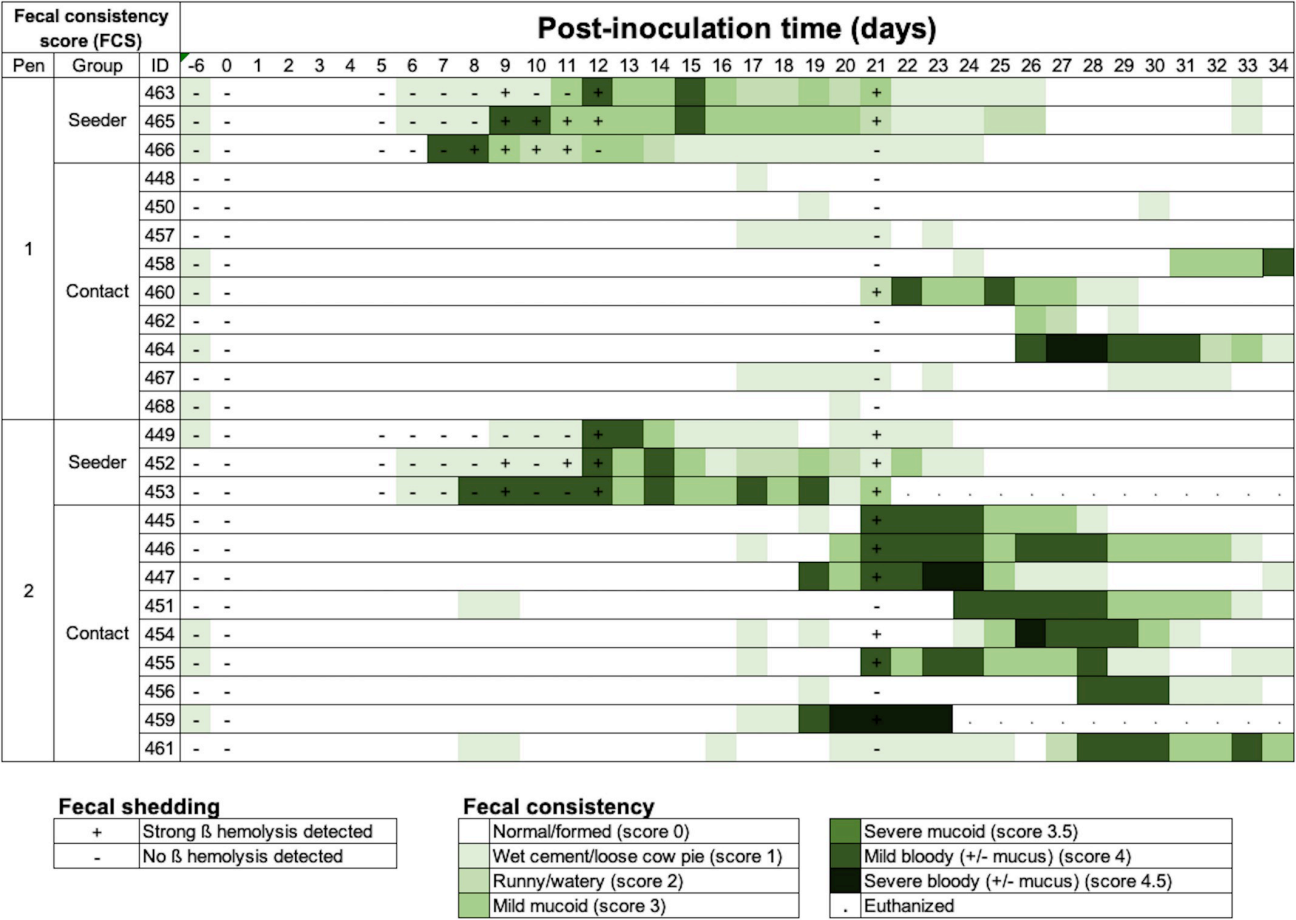
Trial 1 fecal culture and consistency scores (FCS) of seeder and contact pigs. Seeder pigs were inoculated with *B*. *hampsonii* strain 30446 on 0 DPI. Diarrhea severity is represented by an increasing intensity of green: 0 = normal/formed, 1 = wet cement/loose cow pie, 2 = runny/ watery, 3 = mucoid (mild/severe), and 4 = bloody (mild/severe). Fecal shedding was semi-quantified using the culture quadrant technique with positive (strong beta hemolysis) and negative results indicated by (+) or (-), respectively. Note the greater incidence and severity of mucoid or mucohemorrhagic diarrhea in pen 2 compared to pen 1.

**Fig 2:**
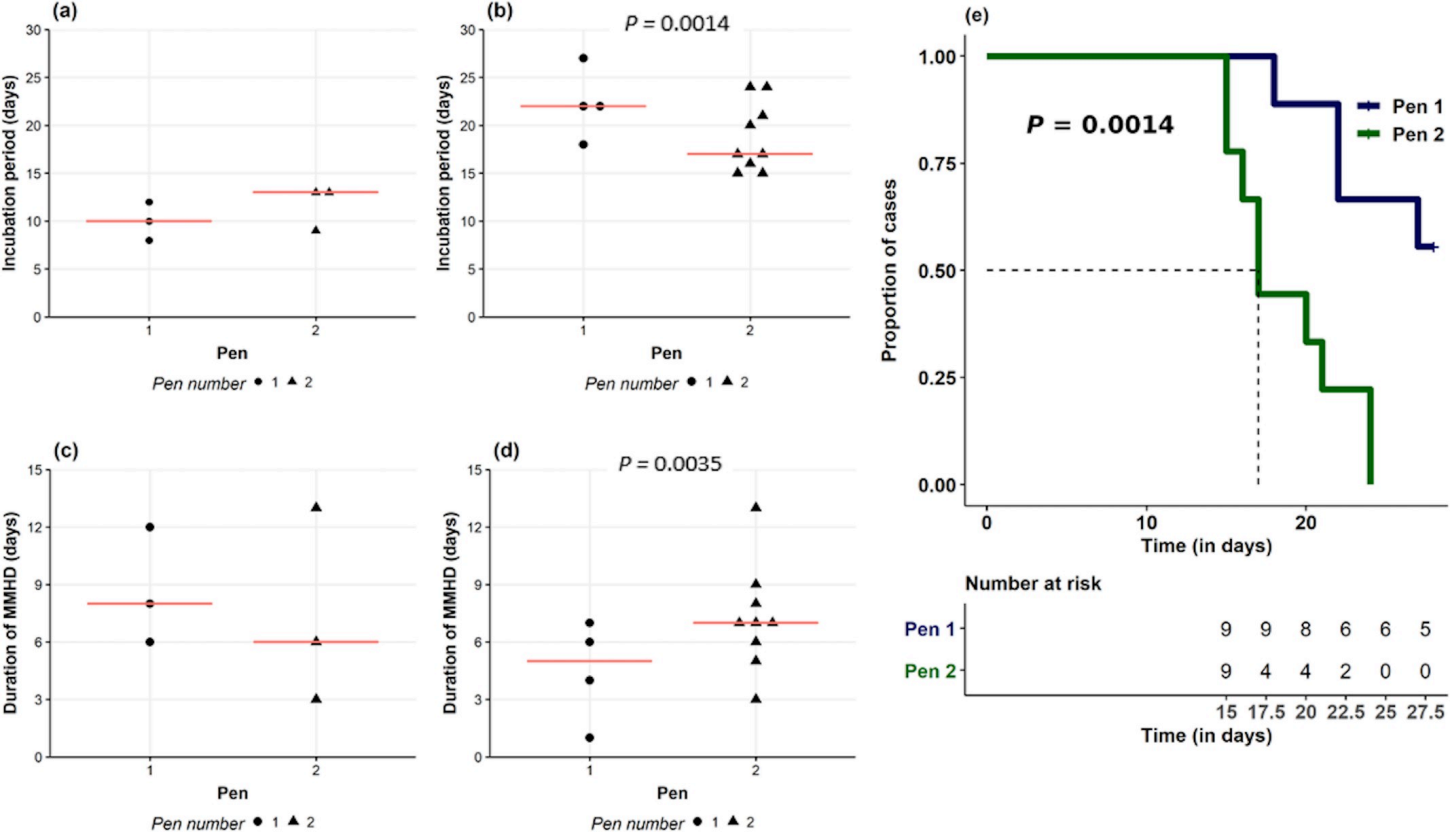
Trial 1 disease incubation, duration and survival in clinically affected seeder and contact pigs. (a) Incubation period in seeder pigs calculated pigs from intragastric inoculation with *B*. *hampsonii* to onset of mucoid or mucohemorrhagic (MMHD), (b) incubation in contact pigs calculated pigs from onset of natural exposure to seeder pigs to onset of MMHD. Duration of mucoid or mucohemorrhagic diarrhea (fecal score ≥ 3) in clinically affected seeder (c) and contact (d) pigs. Median values are showed as a red horizontal line. (e) Kaplan Meier survival curve for time to presentation of MMHD in contact pigs in pens 1 (blue line) and 2 (green line). Each pen contained 9 contact pigs and 3 seeder pigs after regrouping (3:1 ratio). Statistical difference (*P* < .05) is indicated by an asterisk (*).

Following inoculation, all six seeder pigs developed MMHD (Fig 1). The median incubation period was 11 days (range 8–13), median duration of MMHD was 7 days (range 3–12) and median clinical period was 12 days (range 4–14)(S2 Table). One seeder pig (#453) was euthanized on 22 DPI because of decreased body condition score. *Brachyspira* fecal shedding was not detected during the acclimation period but was confirmed in all seeder pigs after inoculation (Fig 1). There was a low but significant correlation (rho = 0.40; *P <* .001) between fecal consistency and culture scores in seeder pigs. There were no pen differences in the incubation period (*P* = 0.2), duration of MMHD (Z = 0.44, *P* = 0.65), clinical period (Z = 0.22, *P* = 0.82) or severity (Z = 0.54, *P* = 0.58) specific to the seeder pig population (Fig 2A and 2C). Except for #453, all seeder pigs recovered to normal fecal consistency (FCS = 0) by 27 DPI without any relapsing before the end of the trial.

In contact pigs (n = 18), the incidence of MMHD was 72.2% (13/18 pigs) (Fig 1). The median incubation period for contact pigs developing MMHD after regrouping with the seeder pigs was 20 days (range 15–27), the median duration of MMHD for clinically affected pigs was 7 days (range 1–13) and the median clinical period for pigs with MMHD was 8 days (range 3–14). Interestingly, five contact pigs never developed MMHD in spite of sporadic observations of FCS = 1, and two contact pigs were still experiencing MMHD at termination. One contact pig (#459) was humanely euthanized on D22 because of prolonged bloody diarrhea with generalized apathy and reluctance to stand. Among the contact pigs, the incidence of MMHD (*P* = 0.03), incubation period (*P <* .01), duration of MMHD (Z = -2.91, *P =* 0.003), clinical period (Z = -.56, *P =* 0.001) and severity (Z = -5.66, *P <* .001) differed by pen, being significantly greater in pen 2 than pen 1 (Fig 2B, 2D and 2E). *Brachyspira* fecal shedding was confirmed in 7/18 (38%) contact pigs at 21 DPI (16 post seeder pig contact). Interestingly, strong hemolysis on the BJ agar cultures was observed in only 1/9 contact pigs (#460) in pen 1 compared to 6/9 in pen 2 (Fig 1).

### Trial 2. B. hampsonii 30446 followed by B. hyodysenteriae G44 natural transmission model using a 1:2 seeder:contact ratio

Pre-screening *Brachyspira* cultures from five random nursery pigs at the farm of origin were negative. During the acclimation period, diarrhea was not observed and *Brachyspira* fecal shedding was not detected by fecal culture, but 6/24 pigs had detected not quantifiable (DNQ) levels of *B*. *hampsonii* 30446 DNA.

**Fig 3:**
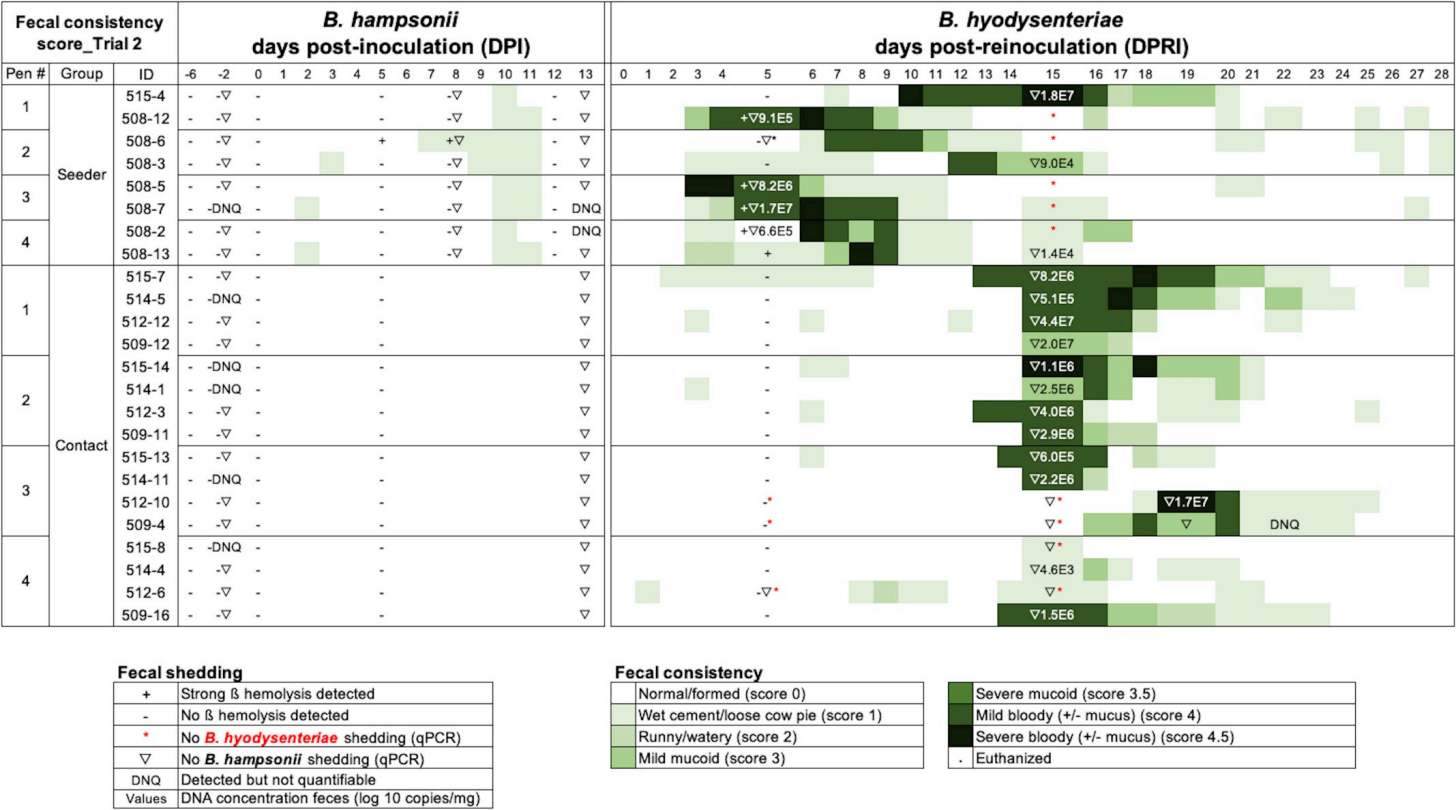
Trial 2 fecal culture and consistency scores (FCS) of seeder and contact pigs. Seeder pigs were inoculated with *B*. *hampsonii* strain 30446 on 0 DPI and reinoculated with *B*. *hyodysenteriae* strain G44 fourteen days later (0 days post re-inoculation (DPR)). Diarrhea severity is represented by an increasing intensity of green: 0 = normal/formed, 1 = wet cement/loose cow pie, 2 = runny/ watery, 3 = mucoid (mild/severe), and 4 = bloody (mild/severe). Fecal shedding was semi-quantified using the culture quadrant technique with positive (strong beta hemolysis) and negative results indicated by (+) or (-), respectively. Presence of *Brachyspira* DNA in feces are represented by symbols: (▽) for *B*. *hampsonii*) or (*) *B*. *hyodysenteriae*. Values in scientific notation are the concentrations of *B*. *hyodysenteriae* DNA in feces. DNQ indicates “detectable target DNA but too low to quantifiable”. Note the lack of mucoid or mucohemorrhagic diarrhea (MMHD) following inoculation with *B*. *hampsonii* and the development of MHD in all seeder pigs after reinoculation with *B*. *hyodysenteriae*.

Culture of fecal samples post *B*. *hampsonii* inoculation from all pigs on 5 DPI and from seeder pigs on 8 DPI unexpectedly resulted in growth of a strongly β hemolytic bacteria with colony formation after 96 hours of incubation in 15/24 (62.5%) and 6/8 (75%) pigs on 5 and 8 DPI, respectively (Fig 3). Subsequent *B*. *hampsonii* (genomovar 2) qPCR performed on hemolytic zones from fecal cultures confirmed the presence of *B*. *hampsonii* 30446 in one seeder pig sample (#508–6). Interestingly, *nox* PCR and species-specific qPCR for *B*. *hampsonii* strain 30446 on 12 DPI feces from all seeder pigs tested negative. None of the seeder pigs developed MMHD anytime up to 14 DPI with *B*. *hampsonii* (Fig 3) and only two seeder pigs (508–7, 508–2) had very low levels (DNQ) of *B*. *hampsonii* DNA in feces on 8 and 13 DPI. For this reason, a decision was made to re-inoculate the same seeder pigs with *B*. *hyodysenteriae* strain G44, which based on experience, was perceived to be more virulent.

**Fig 4:**
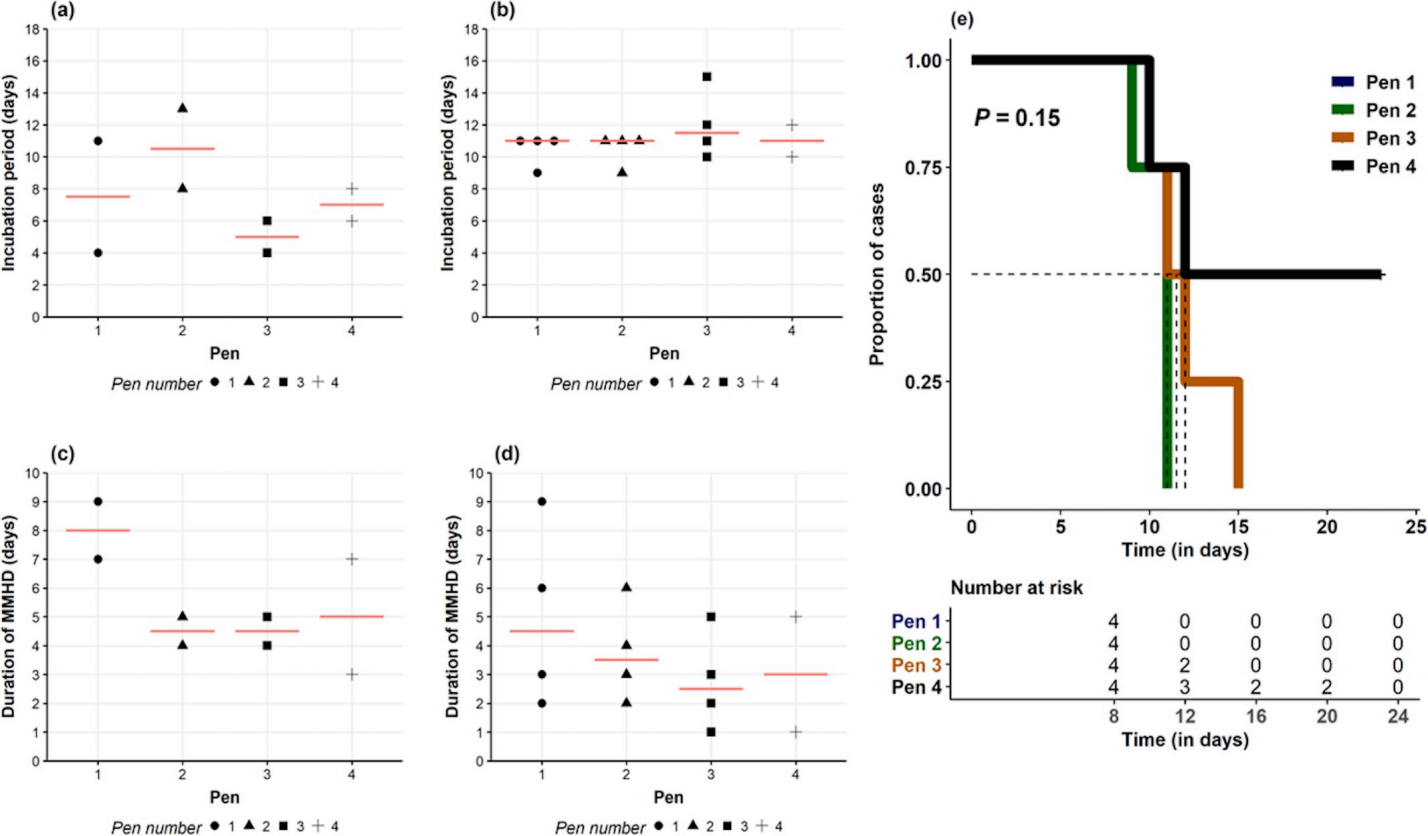
Trial 2 disease incubation, duration and survival in clinically affected seeder and contact pigs. (a) Incubation period in seeder pigs calculated pigs from intragastric reinoculation with *B*. *hampsonii* to onset of mucoid or mucohemorrhagic (MMHD), (b) incubation in contact pigs calculated pigs from onset of natural exposure to seeder pigs to onset of MMHD. Duration of mucoid or mucohemorrhagic diarrhea (fecal score ≥ 3) in clinically affected seeder (c) and contact (d) pigs. Median values are showed as a red horizontal line. (e) Kaplan Meier survival curve for time to presentation of MMHD in contact pigs in pens 1 (blue line), 2 (green line), 3 (orange line), and 4 (black line). Pens 1 and 2 followed the same distribution pattern. Each pen contained 4 contact and 2 seeder pigs after regrouping (2:1 ratio). No statistical pen differences in the duration were observed in either clinically affected seeder (*P* = 0.32) or contact pigs (*P* = 0.21). Note the lower incidence in pen 4 (2/4) when compared with the other pens.

Following re-inoculation with *B*. *hyodysenteriae* strain G44, all seeder pigs developed MMHD (Fig 3) with a median incubation period of 7 days range (4–13), median duration of MMHD of 5 days (range 3–9) (Fig 4A and 4C), and median clinical period of 7 days (range 5–11) (S3 Table). There were no significant pen differences in the incubation period (*P* = 0.28), duration of MMHD (x^2^_3 d.f._ = 3.5, *P* = 0.32), clinical period (x^2^_3 d.f._ = 5.72, *P* = 0.12), or severity of the disease (x^2^_3 d.f._ = 3.85, *P* = 0.27) among the seeder pigs. Interestingly, by 20 DPRI, all seeder pigs had recovered (FCS ≤2) and none relapsed to MMHD during the remaining trial period (Fig 3).

Following reintroduction of the seeder pigs, 14/16 (87.5%) contact pigs developed MMHD (Fig 3). The median incubation period for contact pigs that developed MMHD was 11 days (range 9–15) (Fig 4B) and median duration of MMHD was 3 days (range 1–9) (Fig 4D). The median clinical period for contact pigs with MMHD was 5 days (range 2–10). Two contact pigs (#515–8, 512–6) located in the same pen (pen 4) did not develop MMHD during the entire study despite having a mild diarrhea (FCS 1 or 2) for at least one day. There were no significant differences among contact pen in the incubation period after regrouping with seeder pigs post *B*. *hyodysenteriae* inoculation (*P* = 0.15) (Fig 4E), duration of MMHD (x^2^_3 d.f._ = 4.50, *P* = 0.21), clinical period (x^2^_3 d.f._ = 4.03 *P* = 0.25), or severity of the disease (x^2^_3 d.f._ = 2.79, *P* = 0.42). However, the incidence of the disease across pens showed a marked trend (x^2^_3 d.f._ = 6.85, *P* = 0.07) related to the lower incidence in pen 4 (2/4) compared with the other pens (4/4)(Fig 4E). No significant litter differences were found among contact pigs with respect to the incidence of the disease (x^2^_3 d.f._ = 2.28, *P* = 0.51), incubation period after regrouping with seeder pigs post *B*. *hyodysenteriae* inoculation (*P* = .34), duration of MMHD (x^2^_3 d.f._ = 1.46, *P* = 0.69), clinical period (x^2^_3 d.f._ = 1.82, *P* = 0.60), or severity of the disease (x^2^_3 d.f._ = 1.34, *P* = 0.71).

Five days after reinoculation of the seeder pigs with *B*. *hyodysenteriae*, 4/8 seeder pigs had quantifiable levels of *B*. *hyodysenteriae* in feces (average 6.62×10^6^ GE/g). At 15 DPRI three additional seeder pigs had quantifiable levels (average 5.93×10^6^ GE/g) (Fig 3). *B*. *hampsonii* DNA was not detected in feces by qPCR after *B*. *hyodysenteriae* reinoculation. In contact pigs, quantifiable levels of *B*. *hyodysenteriae* DNA were detected in feces of 13/16 (average 8.05×10^6^ GE/g) on 15–19 DPRI, and very low (DNQ) levels detected in one additional contact pig on 22 DPRI. *B*. *hyodysenteriae* was not detected in the feces of 1/8 seeder and 2/16 contact pigs at any of the sampling time points. One seeder pig (#508–6) experienced MMHD from 7 to 11 DPRI despite negative fecal PCR results before (5 DPRI) and after (15 DPRI). Two contact pigs (#515–8 and 512–6) never developed MMHD. *B*. *hampsonii* was not detected by qPCR in feces from any animal after *B*. *hyodysenteriae* reinoculation.

## Discussion

An ideal natural (seeder pig) transmission model for SD relies on seeder pigs following inoculation to have a high incidence of MMHD, a short incubation period, extended duration of MMHD with a sustained fecal shedding, moderate severity to avoid life-threatening conditions, and consistency of clinical presentation enabling re-grouping with the contacts on the same day to ensure consistency of exposure amongst the contact pigs. Failure to achieve this would lead to greater variation in clinical outcome amongst the contacts which must be compensated for by greater pig numbers and a longer trial duration. In this study, we aimed to optimize a reproducible experimental natural transmission (seeder) model for SD to mimic the natural transmission of pathogenic *Brachyspira* on commercial farms, with the long-term purpose of evaluating the protective efficacy of a vaccine against strongly hemolytic *Brachyspira* strains. Reproducibility is important to ensure consistency among related experiments which in our experience, has been difficult to achieve when inoculating with *B*. *hampsonii*, and has also been reported by Muehlenthaler [16]. For this reason, the seeder pigs in trial 2 were reinoculated with *B*. *hyodysenteriae* strain G44 after failing to develop MMHD within 14 days of intragastric *B*. *hampsonii* inoculation.

A number of experimental animal models have been used to evaluate SD vaccines specific for *B*. *hyodysenteriae* or *B*. *hampsonii* [16–20]. Typical models use direct inoculation by oral administration of various volumes of liquid or solid media where *Brachyspira* growth is quantified using culture or PCR methods. Direct oral bolus inoculation has the advantage of providing more controlled oral doses than a natural transmission model, however, if the oral dose is excessive, it may overwhelm most of natural barriers and immune responses as described for other infectious diseases [21] leading to severe disease harming the animal’s wellbeing or overwhelming a prototype vaccine under evaluation. By contrast, a reproducible, experimental seeder pig transmission model more closely represents the natural infection and disease experienced in grow-finish populations while avoiding some of the uncontrollable factors contributing to variation inherent to commercial field studies. Natural transmission models have been useful in the evaluation of intervention measures for major intestinal diseases in grower pigs including ileitis [22], salmonellosis [23], colibacillosis [24] and SD [25].

The SD infection models using oral and intragastric inoculation of *B*. *hyodysenteriae* have resulted in 100% incidence of disease [17–20]. By contrast, experimental challenge models using *B*. *hampsonii* (genomovar II) by way of three intragastric doses of homogenized agar slurry reported disease incidence rates of 40 to 60% [26–28], and 75% when using pure broth culture [11]. On the other hand, a challenge model inoculation with *B*. *hampsonii* (genomovar I) reported a disease incidence of 66% [29]. In all examples, disease incidence was defined as the development of MMHD and *Brachyspira* shedding during the experimental period.

To our knowledge, only two studies have described natural transmission models for SD. The study of Mahu [9] differs from the present trials by using naturally infected seeder pigs from a commercial source with an acute outbreak of SD instead of intragastrically inoculated seeder pigs. Fecal shedding and clinical SD were reported in 5/14 and 4/14 animals, respectively. Diego [10] used a higher seeder:contact ratio (1:6 versus 1:3 or 1:2) in a vaccine efficacy study and reported fecal shedding in all pigs (unfortunately, the incidence of SD was not reported). Other differences in experimental design between those and the present study include the use of older pigs (7 versus 5 to 6-weeks old pigs) and different *Brachyspira* species and strain (*B*. *hyodysenteriae* strains D28 and B78 versus *B*. *hampsonii* strain 30446 and *B*. *hyodysenteriae* strain G44).

Although the direct intragastric inoculation of *B*. *hampsonii* 30446 (genomovar II) in trial 1 resulted in all seeder pigs developing MMHD and the natural infection of 72% of contact pigs when a 1:3 seeder:contact ratio was applied, the incidence of MMHD in the contact population differed significantly between pens (4/9 in pen 1, 9/9 in pen 2). While these findings may reflect the slow spread of SD in natural outbreaks [30] or other factors influencing susceptibility such as genetic background or response to stress [31], the most compelling explanation relates to a low level of environmental (fecal-oral) exposure resulting in inconsistent transmission to contact pigs. Furthermore, a number of strategies such as group housing, extended fasting, and feeding with high levels of soybean meal have been used or suggested to increase the incidence of MMHD in inoculated pigs [10,25]. In the present trials, all animals were group housed but we purposefully avoided the use of 100% soybean meal (every other day as by Jacobson [25]) because this is not a normal production practice on commercial farms and reduces the external validity of any results related to vaccine efficacy. Similarly, fasting was only used for a limited period (about 15 h) prior to seeder pig inoculation, much shorter than the 72 h period reported by Diego [10].

Given the variation in the incidence of MMHD in contact pigs between pens following *B*. *hampsonii* inoculation in trial 1, trial 2 was designed to further optimize the transmission model. The seeder pig inoculum dose, *Brachyspira* strain, pig farm source, and diet formula were consistent between trials 1 and 2, whereas the group sizes, seeder:contact ratio and pen dimensions were reduced to improve the consistency of transmission from seeder to contact pigs. Unexpectedly, none of the seeder pigs developed clinical signs of SD by 12 DPI after inoculation with *B*. *hampsonii* strain 30446 even though the average inoculation doses of trials 1 and 2 were consistent (trial 1: 8.56 x10^8^, trial 2: 6.97 x10^8^ GE/mL). In trial 2, 6/24 pigs originating from three litters had low (DNQ levels) of *B*. *hampsonii* strain 30446 DNA in feces prior to inoculation (- 2 DPI). Although too low to be clinically important, the results were unexpected and may indicate environmental exposure prior to inoculation. Background immunity related to potential environmental exposure cannot be ruled out and may be the reason why *B*. *hampsonii* intragastric inoculation failed. That being said, this finding has been observed in a previous study [11] where 6/8 pigs with low positive (DNQ) PCR results developed MMHD following inoculation. This suggests the presence of *B*. *hampsonii* 30446 at low levels is not sufficient to cause clinical disease following intragastric inoculation. Nevertheless, verifying the health status of the source farm and pigs used in research is critical and normally requires detailed knowledge of the farm’s history, clinical signs as well as adjunct antigen or antibody testing. While sero-profiling the target population is useful for many diseases, serologic tests are not developed for *B*. *hampsonii* and not available in Canada for *B*. *hyodysenteriae*. Several prototype *B*. *hyodysenteriae* enzyme-linked immunosorbent assays (ELISA) have been developed to detect antibodies circulating in sera [32–34]. An Australian field study [33] showed that seroprevalence varies from <5% to 100% in both reported SD-negative and reported SD-positive farms indicating that serum profiling is most suited for herd-level investigations. Use of serological tests, however, is controversial, as there are numerous instances of non-clinical farms being seropositive but follow-up testing of feces or tissues by culture and/or PCR failed to detect or isolate *B*. *hyodysenteriae* [33]. Moreover, cross-reactions with other *Brachyspira* species is thought to be low based on the heterogeneity of the target amino acid sequence among Brachyspira spp. (70–78% similar) but has not been definitively ruled out. For these reasons, permission for serologic testing of the source farms used would not be granted, and in our view, use for this purpose is not judicious at the present time. Therefore, we employed the best alternative strategy by testing feces from the individual pigs using culture and/or PCR before pre-inoculation. Culture followed by genus specific PCR has the advantage of definitively identifying any *Brachyspira* spp in the sample. This coupled with knowledge of the history, clinical status of the farm and antibiotic use (absence of) is the best strategy available in Canada at this time.

The inoculation failure could also be explained by the lack of sufficient viable *B*. *hampsonii* in the culture broth to cause disease, and highlights the importance of both establishing the minimum effective inoculation dose (organisms per kg liveweight) and confirming the viability of *Brachyspira* in the culture broth prior to inoculation as critical steps to success before other animal or environmental related factors are investigated. After the unsuccessful inoculation with *B*. *hampsonii* 30446 in trial 2, the re-inoculation of the same seeder pigs 14 days later with an average dose of 1.69 x 10^9^ GE/mL of *B*. *hyodysenteriae* strain G44 induced MMHD in all seeder pigs. Although the possibility of virulence differences between *B*. *hampsonii* 30466 and *B*. *hyodysenteriae* G44 seems likely, this is inconsistent with previous results that demonstrated similar virulence between *B*. *hampsonii* strain 30446 and strongly hemolytic *B*. *hyodysenteriae* strain B204 [28] that is closely related genetically to G44 [35]. Despite the initial challenges in this experiment, two important results from this study merit comment. First, the infection of contact pigs showed no biological differences among pens, which suggests the greater consistency of this model using a 1:2 contact:seeder ratio and group sizes of 6 pigs/pen. Second, litter of origin had no effect on biological outcome after *B*. *hyodysenteriae* strain G44 inoculation. In terms of future research, it would be useful to extend the current findings by directly comparing the relative virulence of *B*. *hampsonii* strain 30446 and *B*. *hyodysenteriae* strain G44.

*Brachyspira* spp. do not form colonies on solid agar media. A combination of selective culture where zones of hemolysis are assessed and bacteria are identified by genus-specific PCR and sequencing based on the *nox* gene has shown to be an effective method to speciate *Brachyspira* in feces [36]. In the present study, this combination was used to identify and confirm the presence of *Brachyspira* spp. in the seeder and contact populations. However, the growth of strongly β-hemolytic bacteria with colony formation at 5 DPI in trial 2 complicated the interpretation of fecal culture to monitor ongoing *Brachyspira* shedding in seeder and contact animals. Therefore, the use to culture to assess fecal shedding was discontinued at 5 DPRI. By that time, 4/8 seeder pigs were clinically affected with MMHD. Growth of the strongly β-hemolytic fecal contaminant was unexpected since BJ agar contains several antibiotics (spiramycin, rifampin, vancomycin, colistin, and spectinomycin) to prevent growth of fecal microbiota. Unfortunately, steps to identify the organisms were inadvertently overlooked at the time of the trial and the culture plates were not stored to enable identification after the trial. While there is no way of confirming, a similar examination in a subsequent trial suggested the presence of a mixed bacterial population. Continued efforts to definitively identify strongly β-hemolytic colony forming contaminants will be undertake as the opportunities present.

*Brachyspira* growth can be quantified by estimating absorbance units, qPCR genome equivalents (GE/mL), whole cell counts, and Colony Formation Units (CFU). CFU/mL is the most commonly used metric, but particularly challenging because *Brachyspira* spp. naturally lack colony formation on blood agar. Unfortunately, there is no established consensus among laboratories on how to quantify *Brachyspira* growth or the challenge dose used for direct inoculation. To the best of our knowledge, only two studies have compared quantification methods of *Brachyspira* spp. propagated in liquid media. Muelhenthaler [16] compared whole cell counts, GE, and 50% strong beta-hemolysis endpoint techniques. On the other hand, Kulathunga [37] reported the relationship between CFU/mL, GE, and absorbance units. Such investigations are encouraged as this would lead to better cross-comparison of results among swine dysentery research groups.

## Conclusions

Under the conditions of the trials reported herein, a 1:3 seeder:contact ratio within groups of 12 grower pigs per pen did not consistently sustain natural infection with *B*. *hampsonii* strain 30446 in a susceptible population. However, a transmission model using 1:2 seeder:contact ratio with *B*. *hyodysenteriae* strain G44 for a 4-week exposure period within groups of 6 grower pigs per pen on solid flooring provided sufficient exposure to achieve a natural infection and disease in 14/16 (87.5%) of the contact population with a higher consistency among pens, without the use of excessive amounts of fasting or soybean meal in pre-inoculation diets. However, the failure of intragastric inoculation in trial 2 highlights a frustration when working with *B*. *hampsonii* and indicates future research is needed to improve the consistency of intragastric inoculation.

## Supporting information

S1 Table Ingredient and nutrient specs of the custom Brachyspira #12977 grower diet diets. (DOCX)

S2 Table Trial 1 clinical data summary following inoculation with *B*. *hampsonii*. (XLSX)

S3 Table Trial 2 clinical data summary following re-inoculation with *B*. *hyodysenteriae*. (XLSX)

S4 Table Humane Intervention Point (HIP) checklist. (DOCX)
