# Supplementary material for: Experimental natural transmission (seeder pig) models for reproduction of swine dysentery
Source: PLoS One. 2022 Sep 27;17(9):e0275173. doi: 10.1371/journal.pone.0275173 (PMC9514633; doi:10.1371/journal.pone.0275173)
Supplement: S1 Table — (DOCX) [file pone.0275173.s001.docx]

S1 Table. Ingredient and nutrient specs of the custom Brachyspira #12977 grower diet

| **Ingredient** | **%** |  | **Nutrient** | **%** |
| --- | --- | --- | --- | --- |
| Barley | 25 |  | Crude protein | 16 |
| Wheat | 30 |  | Crude Fiber | 5.6 (8% Max) |
| Corn DDGS | 25 |  | Crude Fat | 3.6 (2% Min) |
| Soybean meal | 10 |  | Digestible Energy | 3120 Kcal |
| Oat hulls | 7.5 |  | Calcium (total) | 0.60 |
| Calcium carbonate | 1.05 |  | Phosphorus (total) | 0.50 |
| Canola oil | 0.5 |  | Sodium | 0.2 |
| Salt (NaCl) | 0.37 |  | Lysine (total) | 0.84 |
| Lysine | 0.25 |  | Methionine (total) | 0.3 |
| Phytase | 0.022 |  | Threonine(total) | 0.62 |
| Micro | 0.31 |  | Tryptophan (total) | 0.19 |
| **Total** | **100.00** |  | Met + Cys (total) | 0.62 |
|  |  |  | Vit A (IU/kg) | 5200 |
|  |  |  | Vit D (IU/kg) | 680 |
|  |  |  | Vit E (IU/kg) | 25 |
|  |  |  | Copper (ppm) | 6 |
|  |  |  | Zinc (ppm) | 100 |
