# Supplementary material for: Experimental natural transmission (seeder pig) models for reproduction of swine dysentery
Source: PLoS One. 2022 Sep 27;17(9):e0275173. doi: 10.1371/journal.pone.0275173 (PMC9514633; doi:10.1371/journal.pone.0275173)
Supplement: S4 Table — (DOCX) [file pone.0275173.s004.docx]

**Humane Intervention Point (HIP) Checklist**

John Harding, Dept. Large Animal Clinical Sciences

**Clinical scoring system: Each inoculated pig will be observed twice daily (before 9 am and after 3 pm) and the clinical signs of disease assessed as follows:** Note: If any clinical sign scores >= 2, medical intervention may be warranted. Please consult Dr. Harding for treatment recommendations.

| **SCORE** | **CLINICAL SIGNS** |
| --- | --- |
| **A: RESPONSIVENESS** | |
| 0 | ALERT AND ACTIVE |
| 1 | ALERT, BUT SLOWER THAN PEN MATES |
| 2 | RELUCTANT TO MOVE, BUT GETS UP BY STIMULATION |
| 3 | DOWN, DOESN’T RESPOND WITH STIMULATION OR DEMONSTRATES SEIZURES (LEG PADDLING, RECUMBENCY, OPISTHOTONOUS) 🡪 *EUTHANASIA* |
| **B: COLOUR OF THE SKIN AND EXTREMITIES** | |
| 0 | NORMAL |
| 1 | SMALL AREA (< 50%) OF SUBCUTANEOUS HYPEREMIA ON THE EAR, TAIL AND ABDOMEN |
| 2 | > 50% OF THE EAR, TAIL OR ABDOMEN HAVE HYPEREMIA OR CYANOSIS, BUT NO OBVIOUS NECROSIS 🡪 *CONSIDER TREATMENT* |
| 3 | > 50% OF THE EAR, TAIL OR ABDOMEN HAVE HYPEREMIA OR CYANOSIS WITH NECROSIS OF THE SKIN 🡪 *EUTHANASIA* |
| **C: RESPIRATION** | |
| 0 | NORMAL |
| 1 | INCREASED RESPIRATION RATE |
| 2 | INCREASED RESPIRATION RATE, SLIGHT ABDOMINAL BREATHING (DYSPNEA) AND/OR COUGHING |
| 3 | INCREASED RESPIRATION RATE AND MARKED ABDOMINAL BREATHING (DYSPNEA) AND/OR PERSISTENT PAROXYSMAL COUGHING 🡪 *CONSIDER EUTHANASIA* |
| **D: CONSISTENCY OF THE FECES** | |
| 0 | FORMED, NORMAL |
| 1 | WET CEMENT OR LOOSE COW PIE |
| 2 | RUNNY OR WATERY |
| 3 | MUCOID |
| 4 | BLOODY OR REDDISH COLOURED (MAY OR MAY NOT BE MUCOID AS WELL)  🡪 *MONITOR DURATION AND* *CONSIDER EUTHANASIA IF >5 DAYS DURATION AND ACCOMPANIED WITH LOSS OF BODY CONDITION AND ROUGH HAIR COAT* |
| **E: BODY CONDITION** | |
| 0 | NORMAL BODY CONDITION AND GUT FILL |
| 1 | NORMAL BODY CONDITION, HOLLOW FLANKS |
| 2 | SLIGHT LOSS OF BODY CONDITION, BACK BONE EVIDENT, WEIGHT LOSS UP TO 15% OF BODY WEIGHT |
| 3 | MODERATE LOSS OF BODY CONDITION, BACK BONE PROMINENT, WEIGHT LOSS GREATER THAN 15% OF BODY WEIGHT 🡪 *CONSIDER EUTHANASIA* |
| **F: BODY TEMPERATURE** – NOT PARTICULARLY RELIABLE IN PIGS AND TAKEN ONLY IF SCORES IN A-E ARE >2 | |
| 0 | 37-39.5⁰C |
| 1 | 39.6-40.5⁰C OR 36-36.9⁰C |
| 2 | 40.6-41.5⁰C OR 35-35.9⁰C |
| 3 | >41.5⁰C OR <35⁰C 🡪 *CONSIDER EUTHANASIA* |
